# Safety, In Vivo Fate, and Degradation of MOF Nanomedicines: Toward Translational Evaluation

**DOI:** 10.3390/pharmaceutics18050548

**Published:** 2026-04-29

**Authors:** Xiaohong Jing, Yuqian Ma, Yi Liu, Xingbin Yin

**Affiliations:** 1 School of Chinese Materia Medica, Beijing University of Chinese Medicine, Beijing 102488, China; 20230935212@bucm.edu.cn; 2 School of Chinese Materia Medica, Tianjin University of Traditional Chinese Medicine, Tianjin 301617, China; m17627809797@163.com

**Keywords:** metal–organic frameworks, safety, in vivo fate, degradation, surface engineering, translational evaluation

## Abstract

Metal–organic frameworks (MOFs) are porous hybrid nanomaterials assembled from metal ions or clusters and organic ligands. Owing to their tunable structures, versatile compositions, and exceptional payload capacities, MOF-based systems have attracted increasing interest in drug delivery and theranostics. Yet, despite rapid progress in efficacy-focused studies, translational evaluation remains limited by incomplete evidence on safety, in vivo fate (pharmacokinetics), and degradation. This review examines MOF nanomedicines through a three-element evidence-chain framework and a four-level material evaluation and substantiation (MES) grading system to relate commonly reported endpoints to development-relevant questions. Major degradation patterns across representative MOF families are summarized, and the influence of surface engineering on safety, in vivo fate, and degradation is discussed. Representative studies are re-examined to illustrate how evidence gaps in these dimensions may affect translational interpretation. Key priorities for the field include dose standardization, quantitative in vivo evaluation, harmonized degradation assays, long-term and repeat-dose studies, and more consistent formulation reporting. By integrating these issues into a unified evidence-chain framework, this review aims to support a more comparable, interpretable, and development-relevant evaluation of MOF nanomedicine research.

## 1. Introduction

Metal–organic frameworks (MOFs) are porous crystalline materials assembled from metal ions or clusters and organic ligands through coordination interactions [1,2,3,4]. Through the tunability of their metal nodes, ligand chemistries, and pore architectures, MOFs can provide relatively exceptional payload capacities, adjustable release behavior, and the potential to combine diagnostic and therapeutic functions within a single platform [1,2,3,4]. These features have supported growing interest in MOF-based systems across several biomedical settings, particularly cancer therapy, antimicrobial treatment, enzyme delivery, nose-to-brain transport, and oral administration [5,6,7,8,9,10,11]. More recently, their reported applications have broadened from systemic delivery to more diverse scenarios, including combination therapy, local hydrogel-based immunotherapy, wound repair, periodontitis treatment, bone-regeneration scaffolds, and microneedle-assisted delivery [12,13,14,15,16,17]. Related studies have also extended to the delivery of nucleic acids and gene-editing tools, local gastric anti-infective and mucosal repair strategies, and cyclodextrin-based MOF systems for oral and food-related delivery [18,19,20,21,22,23]. As MOF platforms move toward more complex in vivo applications, reviews of biological barriers further indicate the need to consider circulation, biodistribution, and barrier interactions in a more integrated manner [24].

**Figure 1:**
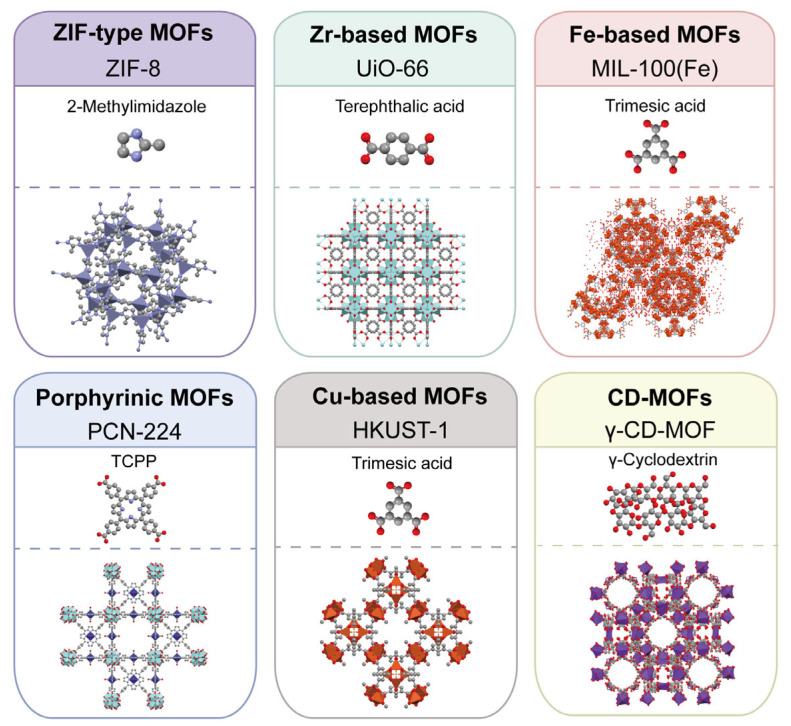
Representative MOF families discussed in biomedical applications and their corresponding ligand motifs. Representative examples are shown for ZIF-type, Zr-based, Fe-based, porphyrinic, Cu-based, and cyclodextrin-based MOFs. The selected examples are illustrative rather than exhaustive and are included to facilitate structural comparison across the major framework classes discussed in the manuscript. Representative framework renderings were generated from corresponding CCDC/CSD crystal structure files using Mercury (CCDC) [30], based on CCDC 2,353,475 (ZIF-8), 2,449,726 (UiO-66), 640,536 (MIL-100(Fe)), 1,550,743 (PCN-224), 943,008 (HKUST-1), and 773,709 (CD-MOF-1). Ligand structures were obtained from PubChem and rendered in Mercury for schematic presentation [31]. Panel colors are used only for visual distinction among MOF families, whereas atom colors indicate different elements in the structural models.

From a structural perspective, MOFs are assembled from metal nodes and organic ligands, and both components jointly influence their physicochemical behavior and biomedical performance [25,26]. Although the MOF family is structurally broad, biomedical and preclinical in vivo-oriented studies have most frequently focused on a more limited group of representative systems, particularly Zr-based frameworks, porphyrinic MOFs, ZIF-type materials, Fe-based MOFs, and selected Cu-based systems such as HKUST-1-related structures [26,27]. These families differ in both metal identity and linker chemistry, which are generally associated with differences in porosity, responsiveness, degradation behavior, and biological interactions [25,28,29]. To facilitate structural comparison across the major framework classes discussed here, representative MOF families and their corresponding ligand motifs are summarized in Figure 1. Representative MOF families relevant to biomedical applications, together with selected compositional features, representative physicochemical features, and translational considerations, are summarized in Table 1.

One challenge in the current literature is not simply the amount of data available, but how unevenly different types of evidence are generated and interpreted. Many studies show that MOFs can load cargo, deliver it, or improve therapeutic performance. Much less often are safety, in vivo fate, and degradation examined in ways that can be read together. This makes direct comparison across studies difficult and limits their translational interpretation. Existing work has nevertheless provided useful clues, including intranasal local release with mucus-associated degradation [42], gastrointestinal stability with limited intestinal passage after oral administration [43,44], and quantitative biodistribution assessed by imaging- or elemental-analysis-based methods [45]. Other studies show how surface engineering can reshape circulation and lesion delivery or how local retention can support sustained release [6,9,16,17,46,47]. Reviews make a similar point: although compatibility-related studies of nanoscale metal–organic frameworks (NMOFs) are increasing, evidence on pharmacokinetic behavior, degradation, and longer-term safety still needs to be integrated more effectively [2,24,40,48].

Against this background, this review takes a translational pharmaceutics and formulation-development perspective. The discussion is organized around a three-element evidence chain: safety, in vivo fate, and degradation. The aim is not simply to ask whether a given MOF platform is mechanistically feasible but whether MOF nanomedicines can be assessed in a way that supports development-oriented judgment.

## 2. A Four-Level MES Grading System for Translational Interpretation

To make the evidence chain more practical, this review introduces a four-level material evaluation and substantiation (MES) grading system. The system is not intended to rank studies by efficacy or publication value. Instead, it indicates how far the available evidence can support development-relevant interpretation. Within this framework, safety, in vivo fate, and degradation are considered together rather than as separate readouts.

At MES Level 1, the evidence is mainly limited to preliminary screening, such as cytotoxicity, hemolysis, basic immune-response assessment, and qualitative observations of structural stability or disassembly in buffered media.

At MES Level 2, the evidence begins to include single-dose tolerability, basic blood biochemistry, gross or histological examination of major organs, qualitative organ distribution, and stimulus-responsive ion release under conditions such as pH or enzymatic challenge.

At MES Level 3, the evidence becomes more relevant to development. It may include short-term multi-organ toxicity with dose context, quantitative biodistribution and pharmacokinetic parameters measured by methods such as inductively coupled plasma mass spectrometry (ICP-MS), and mechanistic degradation in media such as serum or phosphate-buffered saline (PBS) together with drug release.

At MES Level 4, the evidence extends to repeat-dose toxicity, long-term follow-up, immune-toxicity indicators, quantitative excretion through urine, bile, or feces, mass-balance data, and the metabolic fate and long-term retention of both metal components and ligands. The four-level MES grading system is summarized in Table 2.

Importantly, the translational meaning of a given MES level should be considered in relation to the delivery context. This is particularly evident for retention-related evidence, whose significance differs substantially between systemic and local administration. For systemically administered formulations, especially intravenously injected nanomedicines, prolonged in vivo retention generally raises safety concerns because it may indicate incomplete clearance, long-term accumulation, or off-target exposure [48]. By contrast, for locally administered systems, prolonged residence may be functionally desirable rather than inherently problematic, as illustrated by injectable or sustained local-delivery platforms designed to extend local retention or reduce dosing frequency [17]. The MES framework should therefore be understood as a context-aware evidence metric rather than a rigid one-size-fits-all hierarchy.

It should be noted that the MES framework proposed here is a review-specific interpretive framework rather than an official regulatory classification for medicinal products. It is intended to organize the strength and completeness of evidence relevant to translational pharmaceutics, particularly with respect to safety, in vivo fate, and degradation. Real market translation follows a distinct pathway from preclinical evaluation to IND-enabled clinical development, marketing review, and post-approval monitoring [49,50]. The MES levels should therefore be understood as an evidence-interpretation framework, not as a substitute for formal regulatory development stages.

## 3. Distribution of the Evidence Pool Across MES Levels

**Figure 2:**
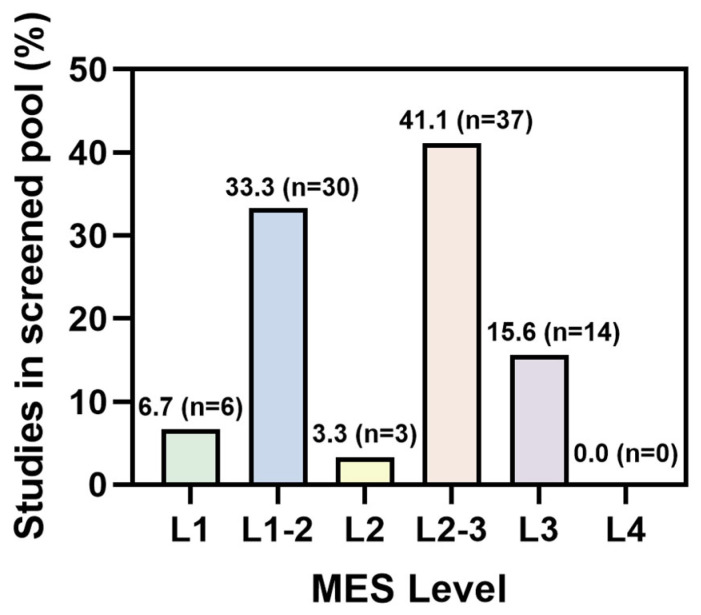
Overview-level percentage distribution of the screened pool of eligible MOF biomedical original studies across MES levels (*n* = 90). Bars show the percentage of studies assigned to the highest MES level clearly supported by the available evidence, and the corresponding study counts are indicated above the bars. This figure summarizes the overall MES distribution of the screened original-study pool, whereas detailed context-sensitive study-by-study interpretation is provided separately in Table S2.

To further characterize how the current literature is distributed across the MES framework, we mapped a screened pool of eligible MOF biomedical original studies to the highest MES level clearly supported by each study. Figure 2 provides an overview-level MES distribution of this screened original-study pool, which was restricted to eligible MOF biomedical original studies that could be assigned an MES level on the basis of the reported evidence. Notably, the pool included not only systemically administered nanomedicine studies but also a subset of contextual local, non-systemic, or mechanistically oriented studies, including scaffold-, hydrogel-, implant-, depot-, and microneedle-based platforms. Because prolonged residence carries different translational implications across these settings, such contextual studies were retained for field-level distribution analysis but were not interpreted as sharing the same translational denominator as systemically administered nanomedicines when discussing clearance-related meaning [51,52,53,54,55,56]. Accordingly, a stricter nanomedicine-oriented study-by-study interpretation was performed separately in Table S2.

Across the screened literature pool, most studies remained concentrated at MES Levels 1–2 or transitional Level 2–3, typically relying on in vitro compatibility screening, short-window tolerability, qualitative organ-associated signals, or simplified stimuli-responsive release. Only a more limited subset reached MES Level 3 by combining stronger in vivo safety with quantitative biodistribution or fate-related analysis and mechanistic stability or degradation testing under physiologically relevant conditions. Representative examples include renal-clearable coordination systems with quantitative fate-related evidence [57], radiolabel- or PET-assisted MOF studies with biodistribution analysis [45,58,59], and selected systemically administered platforms that combine stronger short-term safety packages with fate- or stability-relevant evidence [54,60,61,62]. By contrast, studies approaching MES Level 4 remained rare, largely because repeat-dose toxicity, quantitative excretion, whole-body mass balance, and long-term dual-component fate of both metal nodes and ligands were seldom reported together.

The most common limitation of MES Level 1–2 studies is that safety, in vivo fate, and degradation are often reported separately rather than interpreted as a linked translational evidence chain. Safety evaluation commonly remains limited to cytotoxicity, hemolysis, single-dose tolerability, or short-window histology, whereas in vivo fate is often reduced to qualitative organ-associated signals rather than quantitative pharmacokinetic or excretion analysis. Degradation is likewise often simplified to pH-responsive release or buffered-medium disassembly without mechanistic evaluation under physiologically relevant conditions [63]. By contrast, representative MES Level 3 studies provide a stronger basis for translational discussion but still commonly lack the evidence needed to approach pharmaceutical development more closely, especially repeat-dose toxicity, quantitative excretion, whole-body mass balance, and dual-component fate tracking of both metal nodes and ligands [54,57,59,61,64]. The key limitations at lower MES levels and the remaining gaps in representative Level 3 studies are summarized in Table 3.

## 4. Safety: Toward Interpretable Toxicological Evidence

In MOF-based biomedical research, “good biocompatibility” is a common conclusion and also one of the easiest to overgeneralize. From a translational perspective, safety is not a single endpoint but a layered body of evidence. In the current literature, safety-related evidence most often begins with MES Level 1-type readouts, including in vitro cytotoxicity, hemolysis, and basic immune-activation screening. However, when the full evidence chain of safety, in vivo fate, and degradation is considered, the overall screened study pool is distributed more broadly across transitional categories such as L1–2 and L2–3. Some studies go further and report single-dose tolerability, blood biochemistry, or histological findings from major organs, providing stronger early-stage evidence. However, the translational meaning of such evidence depends strongly on the administration context. For example, the MUV-10 study showed relatively low phosphate-induced degradation, negligible immune-cell toxicity, and minimal immune activation under the tested conditions, supporting an early but comparatively stronger biocompatibility package than simple cytotoxicity alone [65]. By contrast, the PDA-immobilized nano Cu-MOF coating showed favorable blood compatibility in a cardiovascular stent setting, but this type of evidence is closer to the biological evaluation of local implant-associated materials and should not be directly extrapolated to the systemic safety of intravenously administered free MOF nanoparticles [66]. Toxicity-focused reviews further suggest that the toxicological boundaries of MOFs are shaped jointly by metal identity, ligand chemistry, particle size, surface properties, colloidal stability, and degradation products [40,48]. Any conclusion of “low toxicity” therefore needs to be interpreted in the context of the specific formulation and testing conditions.

Safety in MOF-related research is not well captured by the simple statement that a material shows no obvious acute toxicity. A formulation may carry toxicological implications from at least three sources: the intact particle itself, including its size, surface properties, and protein-corona-related interactions; the metal ions released during framework disassembly; and the organic ligands together with their downstream metabolic or transformation products. Original studies increasingly support the view that outer-surface design is integral to safety interpretation rather than a secondary formulation detail. HSA-functionalized Hb@ZIF-8 nanoparticles, for example, showed improved macrophage compatibility, reduced opsonin-related adsorption, and greater colloidal stability in physiologically relevant media [46]. Likewise, DOPA-lipid-bilayer-coated UiO-66 and surface-PEGylated UiO-66 were developed to improve physiological stability and alter uptake-related behavior, underscoring that safety should not be inferred from the “bare” framework alone [64,67]. At the same time, protein adsorption studies and antibody-cloaking strategies further suggest that protein interaction at the MOF interface can reshape biological identity in ways that are relevant to both safety phenotype and in vivo fate [68,69].

Seen in this way, safety in the pharmaceutics context is better described in terms of evidence strength than by asking whether a platform is simply “low-toxic”. Rather than broadly claiming “good biocompatibility”, it is more informative to state what level of evidence has actually been reached. Route-specific in vivo tolerability studies are useful, but their meaning should remain tied to the exposure context. The intranasal ZIF-8 study, for example, showed no significant abnormalities in serum biomarkers, lung diffusing capacity, or tissue morphology after repeated dosing, but its main implication is local mucosal compatibility and prolonged nasal residence rather than systemic clearance or long-term whole-body safety [42]. Similarly, the oral MIL-127 detoxification study demonstrated oral safety, histological protection, strong gastrointestinal stability, and poor intestinal permeation, which are highly informative for a GI-localized oral application but should not be considered equivalent to a systemic nanomedicine safety package [43]. By contrast, studies such as those using intrinsically radioactive 89Zr-UiO-66 achieve a stronger level of evidence because quantitative tracking and safety-related endpoints are considered within the same study [45]. Even so, such findings mainly support feasibility within a defined model and observation window, rather than a conclusion about full long-term translational safety.

Another factor that warrants explicit attention in safety interpretation is the contribution of the organic ligand. Common MOF ligands should not be regarded as inherently biologically neutral by default, because their release and chemical identity can influence how safety findings are interpreted. For imidazole-based systems, particularly ZIF-derived formulations, degradation studies support linker release, and 2-methylimidazole has been shown in rats to be rapidly absorbed and predominantly eliminated in urine, largely as the parent compound [70,71]. Available evidence further indicates that 2-methylimidazole is toxicologically active, although findings from different methylimidazole positional isomers should not be considered directly interchangeable [70,72]. By contrast, direct mammalian toxicokinetic data and standard toxicological evidence for terephthalate-based ligands remain limited in the MOF literature, despite evidence that ligand identity can influence biological responses [73]. Porphyrinic ligands also warrant separate consideration, because porphyrin-related species are biologically active and have been linked to protein binding, oxidative injury, and transporter-mediated homeostasis [74,75,76]. Representative ligand families and their safety-relevant considerations are summarized in Table 4.

## 5. In Vivo Fate: Distribution, Circulation, and Clearance

**Figure 3:**
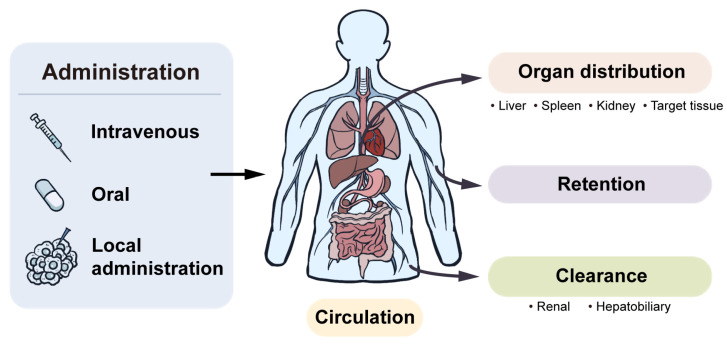
In vivo fate pathway of MOF nanomedicines. Arrows schematically indicate the potential movement and fate-related processes after administration, including circulation, organ distribution, retention, and clearance.

Once MOF nanomedicines enter the body, one of the first questions is where they go. The major fate-related processes after administration, including circulation, organ distribution, retention, and clearance, are schematically illustrated in Figure 3. Across the systemically administered studies discussed here, the liver and spleen are frequent sites of signal detection, whereas kidney-related clearance depends more strongly on particle size, surface hydrophilicity, aggregation state, and surface engineering [45,64,77,78,79,80]. Smaller, better-dispersed, and more hydrophilic systems are more likely to show kidney-associated clearance signals, while larger or less stable systems more often yield stronger liver and spleen signals. Fe-CPNDs provide a similar clue: their ultrasmall hydrodynamic diameter of about 5.3 nm and near-neutral surface were associated with renal-clearable behavior in vivo [57]. This study is particularly informative because it moves beyond simple organ-associated signal detection and begins to link size, colloidal behavior, and elimination route within the same disposition framework [57]. At the cellular level, particle size can also influence downstream fate. In a UiO-66 model, particles of about 150 nm were taken up mainly through clathrin-mediated endocytosis and entered lysosomes to a greater extent, whereas particles of about 260 nm showed combined clathrin- and caveolae-related uptake and were more likely to avoid lysosomal degradation [81]. This observation is relevant because intracellular routing can influence not only local processing but also how degradation, cargo release, and apparent retention are subsequently interpreted.

For translational work, the value of in vivo fate data lies less in detecting organ-associated signals than in obtaining quantitative evidence that can be compared across studies. Quantitative biodistribution and pharmacokinetic analysis bridge efficacy claims and safety interpretation, and imaging-guided PK/biodistribution studies have become an important methodological route in this regard [82]. In the 89Zr-UiO-66/Py-PGA-PEG-F3 system, PET combined with elemental quantification allowed time-dependent distribution, tumor uptake, and clearance trends to be followed within the same analytical framework [45]. In this case, intrinsic radiolabeling and PEG-assisted stabilization enabled PET-based organ distribution analysis together with ex vivo biodistribution and acute/chronic toxicity assessment [45]. Outer-layer engineering can also shift how MOF formulations interact with mucus, blood components, or the tumor microenvironment, thereby changing circulation and exposure patterns [6,7,46,79]. This is illustrated by polymer- or lipid-engineered Zr-MOF systems, in which improved physiological stability and prolonged circulation were accompanied by higher tumor accumulation, showing that fate data should be interpreted together with the surface chemistry that made those distribution patterns possible [64]. Reviews of renal-clearable contrast agents point to size, surface charge, and protein adsorption as key factors in determining whether nanosystems can access the renal elimination route [83]. Yet available studies still vary considerably in how far they cover full pharmacokinetic parameters, mass balance, or long-term retention.

From the perspective of pharmaceutics and development decisions, organ distribution alone is still not enough. What ultimately matters is whether clearance can be demonstrated quantitatively and interpreted in relation to overall disposition. In the oral MIL-127 study, the framework remained largely intact in the gastrointestinal tract, showed limited intestinal passage, and was observed to be excreted in feces [43]. These findings support low systemic exposure, but they do not amount to a complete disposition profile. A related oral study went further by directly examining intestinal crossing of intact MIL-127 and CS-coated MIL-127 nanoparticles, showing that surface chemistry, aggregation behavior, and colloidal stability can influence how much of an orally administered MOF is interpreted as remaining local versus crossing the intestinal barrier [44]. In AP@CS@Lip@HKUST-1, by contrast, the development goal was less to increase systemic exposure than to improve local control while limiting unintended systemic burden, as reflected in inflammatory-site retention, delayed Cu^2+^ release, intracellular *Helicobacter pylori* clearance, and preservation of the intestinal microbiota as far as possible [20]. These route-dependent differences matter because local retention, oral confinement, and systemic circulation cannot be interpreted within a single disposition denominator. A related methodological point also deserves attention. ICP-MS can be a valuable complement for early biodistribution and pharmacokinetic assessment, but it fundamentally tracks elemental signals rather than intact molecular species. Its interpretation therefore still depends on chemical speciation and label stability [84]. Accordingly, elemental biodistribution, radiotracing, and material-stability information are most informative when interpreted together rather than as interchangeable readouts of intact-carrier fate.

## 6. Degradation: Fate After Framework Disassembly

If in vivo fate addresses where a MOF goes, degradation addresses what it becomes. For MOFs, degradation is not a secondary issue. It shapes safety, release behavior, and longer-term exposure. Different MOF families also behave differently in biological media, depending on their metal nodes, ligand structures, and surrounding conditions. ZIF-8 and related systems remain relatively stable under neutral conditions but disassemble more readily in acidic environments. This property has been widely used in systems designed for tumor therapy, infection-related delivery, and the transport of proteins or nucleic acids [8,10,11,42,47,60,85,86,87]. Conversely, some oral formulations are designed to remain more stable in gastrointestinal environments in order to limit premature collapse and systemic absorption [6,43]. Other platforms follow different patterns. MUV-10, for example, showed relatively low phosphate-induced degradation in phosphate-buffered media compared with several commonly discussed MOF systems [65]. Surface engineering can further change these behaviors, because PDA coatings, protein functionalization, and membrane-based shells may alter media access and ion exchange within the framework [8,9,46,47,85]. Original surface-engineering studies further support this point: selective PEGylation of UiO-66 improved phosphate stability, whereas HSA functionalization of Hb@ZIF-8 altered colloidal behavior in biologically relevant media [46,67]. Comparable behavior is also seen in non-classical systems, including Mg2(olz), KBM-1/KBM-2, iron–fumarate nanoparticles, and some silica–MOF or Zr-MOF platforms [18,19,21,88,89]. Reviews in the field also describe physiological stability and target-site degradability as a central design tension [2,3,40].

Still, many degradation studies remain at the level of structural disruption or ion release. In many ZIF-based systems, acid-triggered release or framework disassembly under specific conditions can already be shown [8,10,11,60,87]. What remains much less clear is the later chemical form and in vivo fate of the released metal nodes and ligands. For this reason, terms such as “biodegradable” or “metabolizable” should not be taken as evidence of safe clearance unless they are supported by time-resolved tracking and species-level identification [12,90,91]. Many intracellularly responsive delivery systems therefore provide evidence for endosomal or lysosomal release rather than for a complete in vivo degradation–excretion pathway. More recent work also suggests that degradation can be more complex than gross structural change alone. In situ AFM showed that erosion of iron carboxylate MOFs in PBS depends on surface defects and medium conditions, and visible crystal dimensions do not necessarily change in parallel [63]. A framework-exchange strategy in core–shell MOFs similarly suggested stepwise degradation in tumor-related microenvironments but only in specific systems under specific conditions [92]. Together with broader degradation studies across different MOF families, these findings indicate that degradation should not be reduced to a binary judgment of “intact” versus “collapsed”.

For translational interpretation, however, degradation should not be evaluated only in simplified acidic or buffered systems. For nanoparticle formulations, an additional minimum layer of evidence is whether the material remains chemically and colloidally stable under physiologically relevant ionic and protein-containing conditions because cargo leakage, aggregation, protein adsorption, and protein-mediated dissolution may all alter how subsequent cell-based findings are interpreted. Useful test environments may include saline- or phosphate-containing media across relevant pH conditions, serum-containing cell culture media, albumin solutions, and serum- or plasma-relevant media when feasible. Recent studies likewise show that colloidal behavior in cell medium can be formulation-dependent [93], that protein adsorption at MOF surfaces varies with surface properties [68], and that cargo leakage from ZIF-8 in blood-mimicking media can be strongly albumin-dependent and may become substantial in certain nanoparticle formulations [94]. Taken together, these representative studies show that biologically relevant degradation evidence requires more than pH-triggered release alone and is best interpreted alongside colloidal stability, protein interaction, and cargo-leakage behavior.

Representative original studies and their key translationally relevant findings across safety, in vivo fate, and degradation/stability-related evaluation are summarized in Table 5.

## 7. Convergence of Safety, In Vivo Fate, and Degradation

**Figure 4:**
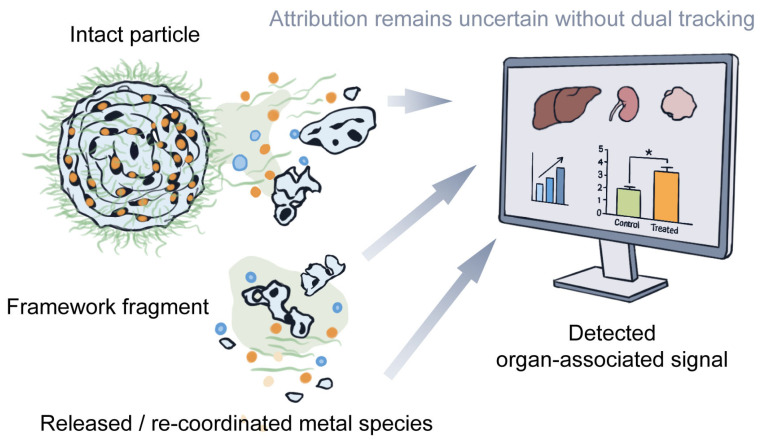
Possible interpretations of an organ-associated signal in MOF nanomedicine studies. Arrows schematically indicate possible contributions of intact particles, framework fragments, and released or re-coordinated metal species to the detected organ-associated signal. Colors are used only for schematic distinction. The asterisk in the illustrative bar chart is schematic and does not indicate statistical significance.

The three-element evidence chain comes together most clearly in questions of clearance and longer-term retention. Only when in vivo fate and degradation are considered side by side can an organ-associated metal signal be interpreted with more confidence—whether it reflects an intact particle, a framework fragment, or a released or re-coordinated metal species, as schematically illustrated in Figure 4. By the same logic, “degradable” remains only a partial description unless the fate of the degradation products is also followed. Quantitatively tracked systems illustrate why this matters, although overall mass-balance judgments still require caution if ligands or other outer-layer components are not tracked at the same time. Representative original studies summarized in Table 5 further show that stronger evidence rarely comes from safety, fate, or degradation data in isolation but rather from partial convergence across these domains. For example, radiolabel-assisted systemic studies such as 89Zr-UiO-66 improve interpretability because distribution, circulation, and at least some safety-related endpoints are assessed within the same analytical path, yet even these systems do not fully resolve the later chemical fate of all framework-derived components [45].

This is also one of the most common interpretive limits in the studies discussed here. Many reports can already show stimulus-triggered disassembly or time-dependent organ distribution. What they less often show is how those signals relate to the intact formulation itself. Without a dual-tracking strategy—for example, tracing both the metal node and the organic ligand—it remains difficult to decide what the detected signal actually represents. Radiolabeling, ICP-MS-based quantification, and multi-time-point excretion analysis are especially useful at this stage. At the same time, recent mechanistic studies make clear why this distinction matters: direct cargo-leakage measurements in physiological media show that released payload does not necessarily report the intact fate of the carrier [94], while in situ AFM has shown that framework erosion can proceed without obvious gross particle collapse [63]. Route-dependent systems reinforce the same point from a different angle. In oral MIL-127 studies, low intestinal passage and route-specific confinement were as important to interpretation as the material’s intrinsic stability, because local retention and systemic circulation do not carry the same translational meaning [44]. Even then, only a small number of studies come close to a relatively complete distribution–degradation–excretion evidence loop. As a result, many positive conclusions in the current literature are more appropriately understood as support for further investigation than as proof of full developability. In this sense, the practical value of the MES framework is not that it replaces formal development stages but that it helps distinguish studies that are merely promising from those that are becoming genuinely interpretable for translational pharmaceutics.

## 8. Surface Engineering Across Safety, In Vivo Fate, and Degradation

**Figure 5:**
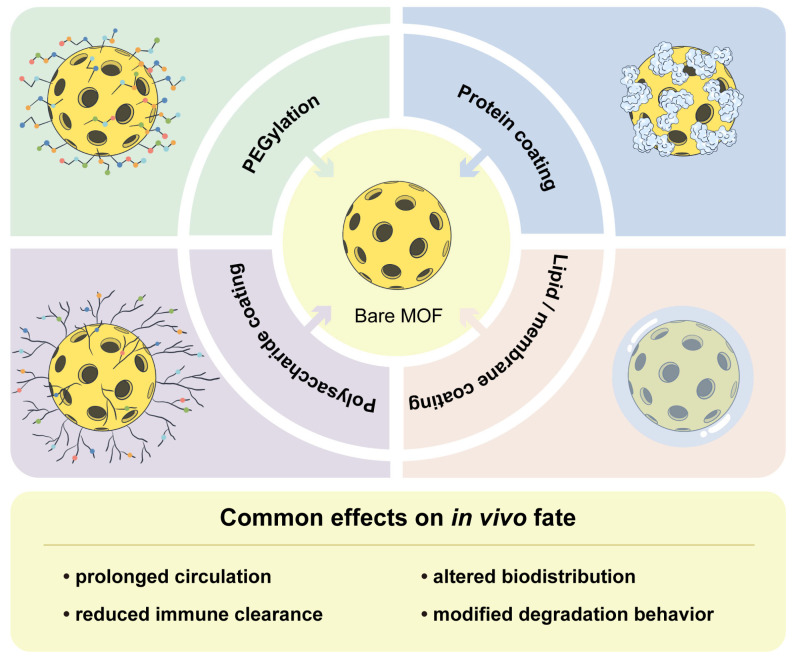
Surface engineering strategies for MOF nanomedicines and their common effects on in vivo fate. Arrows schematically indicate the transition from bare MOFs to surface-engineered formulations, and panel colors are used only for visual distinction among different coating strategies.

Surface engineering matters in MOF nanomedicine not only for targeting or formulation stability but also for the way it can reshape safety, in vivo fate, and degradation. Interface designs ranging from proteins and polymeric shells to membrane-based outer layers have been used to modify how MOFs interact with biological environments [6,7,8,9,10,46,47]. More recent studies extend this range to lipid, polysaccharide, and cell-membrane coatings [13,64,77,78,79,80,90]. Reviews on surface functionalization also highlight recurring aims such as reduced nonspecific protein adsorption, improved circulation, better access to the tumor microenvironment, and enhanced cellular internalization [24]. Reviews of cell-membrane-coated porphyrinic NMOFs point to similar possibilities, including prolonged circulation, reduced reticuloendothelial clearance, and improved tumor targeting or photodynamic therapy (PDT) performance, while also indicating that these effects are not equivalent across membrane sources [95]. Representative original studies support this broader view. HSA-functionalized Hb@ZIF-8 improved colloidal stability and reduced opsonin-related adsorption, DOPA-lipid-bilayer-coated UiO-66 improved physiological stability together with circulation behavior, and surface-PEGylated UiO-66 altered both phosphate stability and uptake-related interpretation [46,64,67]. Representative surface-engineering strategies and their common effects on in vivo fate are summarized in Figure 5.

Notably, responsive disassembly observed in simplified buffered media may not directly predict behavior in complex biological fluids. Once MOF nanomedicines enter protein-rich environments, albumin and other serum proteins can adsorb onto the particle surface and form a biomolecular corona, thereby altering surface accessibility and subsequent biological interactions [96,97,98]. Albumin deserves particular attention in this context because it is a major blood protein capable of interacting strongly with metal-containing MOF surfaces and thereby reshaping the biological identity of the formulation. In MOF systems, both particle features and ligand chemistry can influence protein binding and corona composition [98]. Such corona formation may, in turn, influence circulation persistence, bloodstream half-life, biodistribution, and cell-level interactions. These observations suggest that pH- or enzyme-responsive disassembly demonstrated in buffered media may be attenuated or otherwise modified under biologically relevant conditions. For pharmaceutics-oriented evaluation, degradation and responsiveness should therefore be interpreted alongside data obtained in serum-containing or other biologically relevant media whenever possible [97]. Accordingly, it is useful to report not only stimulus-responsive release but also basic colloidal-stability readouts under these conditions, because surface engineering itself may reshape dispersion, stability, and protein-adhesion behavior in biological environments [69,93,94,99]. This point is reinforced by direct original studies. Protein adsorption on nanoMOF surfaces varies with both particle architecture and protein type [68], antibody precoating of MOF-808 has been used to reduce subsequent protein adhesion [69], and PEG-mediated mineralization of ZIF-8 improved dispersity and stability in cell medium [93]. Complementary cargo-leakage measurements further show that release from ZIF-8 in blood-mimicking media can become strongly albumin-dependent, further indicating that protein-rich environments may reshape how surface engineering affects subsequent biological interpretation [94].

Hydrophilic polymers and protein-based coatings are among the most common strategies. Albumin-functionalized Hb@ZIF-8 and PDA-coated ZIF-8 suggest that the outer layer may improve dispersion and reduce nonspecific interactions while also changing blood compatibility, medium stability, and trigger conditions for release [46,85]. Selective surface PEGylation of UiO-66 further showed improved phosphate stability at pH 7.4, reduced burst release, and altered uptake behavior [67]. DOPA lipid layers, HA, polysaccharides, and membrane-based coatings can also influence local retention, uptake pathways, and microenvironmental accessibility [64,77,78,79,90]. Reviews on cyclodextrin and related materials also place CD-MOFs alongside liposomes and emulsions as delivery modules, suggesting that interface–carrier co-design may shape how cargos are presented and released [22]. In oral systems, this same logic is seen in chitosan-coated MIL-127, where the outer layer influenced colloidal stability, chemical stability, and intestinal crossing behavior together rather than separately [44].

Biomimetic membrane coating provides another example of this three-way regulation. Cell membranes and related outer layers can reduce nonspecific recognition, improve lesion accumulation in some models, and introduce additional biological interactions in settings such as infection, cancer, or enzyme replacement [8,9,47]. In more recent studies, red-blood-cell-membrane-coated TGZ@eM and tumor-cell-membrane-coated AQ4N/GOx@ZIF-8@CM were both used to obtain immune-evasive and prolonged-circulation features [77,78]. A fusion-membrane-modified Fe-TCPP platform further combined membrane-mediated immunomodulation with ferroptosis and photodynamic effects in one system [90]. These potential advantages, however, are accompanied by greater immunological uncertainty and more demanding formulation characterization. Membrane-protein fidelity, coating density, shell stability, endotoxin control, and batch consistency may all influence how safety is interpreted, while also affecting in vivo fate and degradation. Functional outcomes alone may therefore be insufficient to support broader translational claims. Original biomimetic studies reinforce both sides of this trade-off. Erythrocyte-membrane-cloaked MOF systems have been used to obtain immune-evasive and prolonged-circulation features [77], while other biomimetic outer layers have been designed to combine targeting, microenvironment interaction, and therapy in one platform [78]. Even so, these formulations remain more demanding to characterize than relatively simple polymer- or lipid-based surface modifications, because membrane source, coating integrity, protein retention, and batch reproducibility all affect how safety, fate, and degradation should be interpreted.

For this reason, surface engineering should not be described only as a way to improve efficacy. From a translational pharmaceutics perspective, it also belongs within formulation and chemistry, manufacturing, and control (CMC) discussions. Particle size and polydispersity index (PDI), surface charge, coating density, residual solvent, sterilization compatibility, storage stability, and freeze-drying feasibility may all influence the three core types of evidence through protein-corona formation, opsonization, medium penetration, and framework erosion. However, surface-engineering strategies are not equivalent when considering manufacturability. Compared with relatively simple and well-defined modifications such as PEGylation or selected polymer/lipid coatings, biomimetic approaches like cell-membrane coating may introduce greater challenges in source-material standardization, membrane preparation and coating procedures, structural and functional characterization, batch consistency, and regulatory interpretation [100,101]. Although cell-membrane coating can offer biologically attractive multifunctionality, current translational analyses of nanomedicines continue to identify large-scale production, reproducibility, and clinically relevant evaluation frameworks as key barriers to broader development [100,102]. Surface engineering should therefore be evaluated not only by whether it improves circulation or targeting but also by whether the resulting formulation remains manufacturable, characterizable, and scalable under conditions relevant to development.

## 9. Representative Cases and Evidence Completeness

Applying the three-element evidence-chain framework to specific studies helps clarify where each case is informative and where its limits remain. In the oral detoxification study of MIL-127, the main value was not greater systemic exposure but a safety-oriented design based on gastrointestinal stability, limited intestinal passage, and fecal excretion [43]. The intestinal-crossing study points to the same issue from another angle: even when intact MOF transport across the intestinal barrier can be demonstrated, oral administration should not be assumed to ensure efficient systemic delivery [44]. In local-delivery and tissue-repair settings, F-HKUST-1 used controlled local Cu^2+^ release to support wound healing [13], whereas FeCu-MOF scaffolds and microneedle systems were developed more clearly for tissue engineering or sustained local administration [15,16]. In oral and gastrointestinal delivery, γ-CD-MOF combined with micelles improved the apparent solubility and bioaccessibility of curcumin under in vitro digestion conditions [23]. This also highlights a broader point: after MOF disassembly, a released hydrophobic guest may not remain in a soluble or readily available form.

Related formulation-engineering studies point in the same direction. Systems such as MPDA@ZIF-8/DOX+GOx, Van@ZIF-8@PDA, and ZIF-90 show how stability, triggered release, and therapeutic effect can be tuned together [10,60,87]. In situ polymer coating can improve physiological stability while preserving intracellular responsiveness [61], while PIC/CA4@ZIF-8/HA hydrogel extends local retention and reduces dosing frequency [17]. Other platforms also use acid-triggered or dual-triggered mechanisms to regulate therapeutic cargo release [103,104,105]. Without corresponding data on clearance, longer-term exposure, or repeat dosing, however, these studies still speak more clearly to mechanistic feasibility than to full translational readiness.

Some studies are more informative because they place several types of evidence within the same experimental path. In 89Zr-UiO-66/Py-PGA-PEG-F3 nanodots, stable labeling, quantitative biodistribution, and clearance trends were considered together [45]. Other biomimetic and membrane-engineered systems move in a similar direction by combining interface design with in vivo distribution and safety-related evaluation [12,90,106]. Their value lies not only in functional outcomes but also in relating those outcomes to a fuller in vivo disposition logic.

## 10. Standardization Priorities for Translational Pharmaceutics

**Figure 6:**
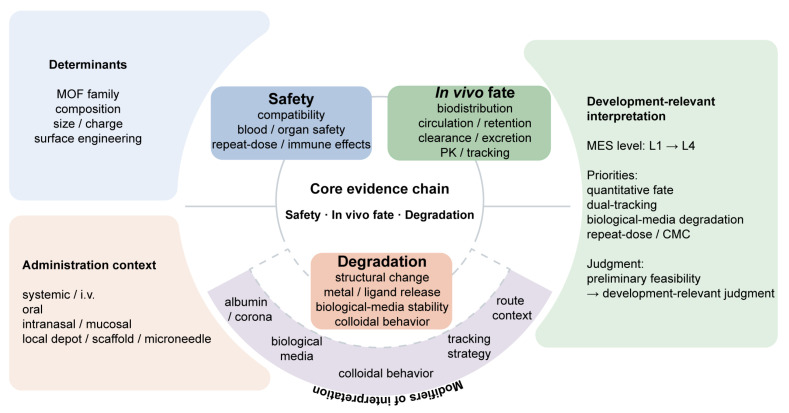
Framework for development-relevant evaluation of MOF nanomedicines. Solid arrows indicate the main evidence-flow logic, whereas dashed lines denote contextual modifiers of interpretation.

To synthesize the major concepts discussed throughout this review into a development-relevant framework, the overall logic linking formulation determinants, administration context, the three-element evidence chain, and development-relevant interpretation is summarized in Figure 6.

If MOF nanomedicine research is to move closer to translational use, both reporting and study design need to become more consistent. Cross-study interpretation is often shaped by recurrent variables such as framework type, particle size, surface chemistry, administration context, protein interaction, and tracking strategy, which help explain why apparently similar biological findings may carry very different translational meanings. One basic issue is dose normalization. In addition to mass dose (mg/kg), metal molar dose is best reported whenever possible, together with formulation concentration, administration volume, route, and dosing frequency. Because different frameworks can contain very different amounts of metal, mass-based dosing alone often limits cross-study comparison. At the same time, although potency-related metrics such as IC_50_ may be informative in selected contexts, direct cross-study comparison remains difficult in the current MOF literature. Reported systems vary substantially in framework composition, surface engineering, payload type, administration route, irradiation or trigger conditions, biological model, and endpoint design. As a result, even when IC_50_-like values are available, they do not necessarily reflect comparable exposure logic or translational significance across studies. For this reason, the present review emphasizes representative dose and exposure context rather than implying strict quantitative equivalence across heterogeneous MOF platforms.

A second priority is a more quantitative evaluation of in vivo fate. For metal nodes, ICP-MS can serve as a practical baseline tool and, where feasible, can be complemented by radiolabeling or other calibrated approaches for multi-time-point distribution and excretion analysis. Quantitative radiotracing studies suggest that time-linked distribution and clearance data are more informative for development decisions than simply detecting where signals appear. A recent methodological perspective on ICP-MS makes a related point: elemental quantification is useful in early distribution and release studies, but it does not by itself preserve full chemical-species information [84].

These limitations highlight the need for more component-resolved analytical strategies. Radiolabeling-based approaches already provide a practical route toward stronger evidence at MES Level 3–4 by enabling time-resolved biodistribution and clearance analysis in vivo. In MOF-related systems, representative studies have shown that framework-associated radiolabeling can quantify circulation, organ distribution, and clearance profiles and can, in some cases, be integrated with stability and toxicity assessments within the same workflow [58,59]. A further step would be to adopt dual-tracking strategies that separately follow the metal node and the organic component. In a broader nanocarrier context, dual radiolabeling has provided proof-of-principle that different components of a multicomponent nanosystem may not remain associated in vivo and may therefore exhibit distinct biodistribution profiles [107]. By extension, this strategy could help distinguish framework integrity from post-disassembly fate in MOF nanomedicine. LC-MS/MS-based quantification of released ligands or their metabolites may serve as a complementary approach for clarifying the fate of the organic component. At present, however, such ligand-resolved approaches should be regarded as an important methodological direction rather than an established routine standard in the current MOF nanomedicine literature.

Degradation studies also need more comparable conditions. Reports should define phosphate concentration, serum fraction, pH, and temperature as clearly as possible and should ideally follow structural change, metal-ion release, and ligand release over time. At a practical level, this may be approached by combining colloidal readouts such as hydrodynamic size, polydispersity index, zeta potential, turbidity, or sedimentation rate with structural and component-related release measurements. In selected probe-loaded systems, continuous-wave EPR can further provide direct quantitative tracking of cargo leakage in physiological media [94]. This is especially relevant for acid-responsive ZIF systems, oral formulations designed for gastrointestinal stability, and frameworks that differ in phosphate sensitivity [8,10,11,42,43,60,65]. An in situ AFM study further showed that initial surface defects and phosphate or acid–base conditions can jointly affect erosion behavior in iron carboxylate MOFs [63]. More broadly, reviews of two-dimensional nanomaterials suggest that biodegradation assessment benefits from considering redox conditions, enzymes, pH, and cellular environments together [108].

Longer follow-up and repeat-dose studies would be valuable on a more routine basis. For platforms intended for multi-cycle treatment, chronic use, or repeated exposure, a 24–48 h window after a single dose is rarely enough to support translational judgment. Even when some studies already show reduced dosing frequency, prolonged local retention, or no obvious toxicity within the observed period [12,17,90], repeat-dose toxicity, extended follow-up, and appropriate immune-toxicity panels remain important parts of higher-level evidence.

Another practical point is the tracking strategy. Tracking the metal node alone, or observing framework collapse without following released components, can leave clearance and retention open to overinterpretation. Wherever possible, a dual-tracking approach is therefore worth considering.

Finally, formulation and CMC reporting also need to be more consistent. Batch-to-batch particle size and PDI, surface-modification density, endotoxin levels, residual solvent, sterilization method, and storage stability can all affect the reproducibility and comparability of in vivo fate and safety data, especially in membrane-coated, sugar-layer-modified, or protein-functionalized systems [6,7,8,9,46,47]. Mild preparation strategies such as biomineralization-based assembly also suggest that process conditions may influence developability [105]. Reviews on MOF-based drug delivery and tumor therapy continue to identify scale-up, quality control, nomenclature, and regulatory communication as practical barriers to further development [2,3,4], while nucleic-acid-delivery reviews further highlight protein corona effects, endosomal escape efficiency, batch consistency, and longer-term biosafety as translational constraints [109].

## 11. Limitations and Outlook

MOF platforms differ substantially in metal nodes, ligand structures, surface engineering, indications, animal models, and analytical methods. The discussion here is therefore intended less as a quantitative comparison of platform performance than as a way to organize how evidence chains may be built. The studies covered intranasal, intravenous, oral, local injection, microneedle, and scaffold-based settings, as well as a wide range of platforms, including ZIF, UiO, MIL, coordination-polymer nanodots, membrane-coated MOFs, MOF-based composite scaffolds, CD-MOFs, and Bio-MOFs. This heterogeneity itself makes direct cross-study extrapolation difficult, as also reflected in recent reviews from the perspectives of drug delivery, imaging, surface functionalization, and safety [1,2,3,4,24,40,41]. It is especially apparent in bone-related MOF systems, where study endpoints can differ markedly across disease settings and application contexts [110]. At the same time, the current literature has already established that MOF platforms can achieve functional delivery and therapeutic effects across diverse administration settings and that a limited subset of studies has begun to combine stronger safety, fate, and degradation evidence within the same experimental path. What remains less developed is the generation of sufficiently integrated and comparable evidence to support development-oriented interpretation. In practice, the most persistent barriers are the lack of quantitative fate and excretion logic, incomplete dual-component tracking, insufficiently standardized degradation testing in biologically relevant media, and inconsistent formulation and CMC-related reporting across studies.

Seen from this perspective, the three-element evidence-chain framework and MES grading system proposed here are better understood as working models for organizing and interpreting the literature, rather than fixed classifications. As more studies begin to include quantitative excretion, long-term retention tracking, ligand-metabolism analysis, and more standardized reporting of formulation parameters, the framework itself should also be revised and refined. This is likely to be particularly important for systems such as local-delivery platforms, tissue-engineering materials, and vaccine formulations, where exposure patterns differ from those of more conventional intravenously administered nanomedicines. Reviews in tumor- and nucleic-acid-delivery fields point to the same issue: although studies on the in vitro and in vivo compatibility of NMOF- and MOF-based systems are increasing, mechanism, pharmacokinetic behavior, longer-term biosafety, and manufacturing consistency continue to constrain translational interpretation and clinical development [2,3,4,37,40,48,109].

## 12. Conclusions

MOF nanomedicine research has generated substantial evidence that functional effects can be achieved. What remains more limited is integrated evidence that can support development-oriented judgment. For coordination-based nanomaterials that undergo structural transformation in vivo, safety, in vivo fate, and degradation need to be interpreted within the same translational framework rather than treated as three isolated topics. The studies reviewed here suggest that the translational meaning of apparently similar conclusions—such as “good biocompatibility” or “responsive degradation”—can differ substantially depending on the route of administration, outer-layer engineering, depth of quantitative tracking, and completeness of longer-term evidence. Accordingly, progress in this field will depend not only on accumulating additional efficacy-focused studies but also on building more integrated evidence packages that connect route-aware safety assessment, quantitative biodistribution and clearance analysis, and degradation testing under biologically relevant conditions. In practice, it may be more useful to ask not whether a given MOF platform is simply “safe” or “degradable” but under what conditions, with what level of evidence, and to what extent it can support a development-relevant judgment. More comparable dose normalization, stronger repeat-dose and longer-term study designs, clearer tracking of framework-derived and cargo-related components, and more consistent formulation and CMC-related reporting should help move the field toward a more interpretable and development-relevant path forward.

## Figures and Tables

**Table 1:** Representative MOF families relevant to biomedical applications, selected compositional features, representative physicochemical features, and translational considerations.

MOF Family	Representative Framework(s)	Representative Metals	Typical Ligand Motifs	Representative Physicochemical Features	Selected Biomedical Relevance	Key Considerations	References
ZIF-type MOFs	ZIF-8, ZIF-67, ZIF-90	Zn, Co	Imidazolate/2-methylimidazole and related imidazolate linkers	Microporous frameworks with characteristic cage-aperture architecture; biomedical formulations are commonly nanosized, whereas particle size and zeta potential remain highly synthesis-, coating-, and medium-dependent	Widely explored in drug delivery, biomineralization, cargo protection, and pH-responsive systems; compatible with relatively mild synthetic conditions	Strongly environment-dependent dissociation and physiological instability should be considered; ion/linker release and protein- or buffer-mediated decomposition may affect biological interpretation	[27,28,32]
Zr-based MOFs (including UiO-type systems)	UiO-66, UiO-67, UiO-66-NH2, MOF-808	Zr	Terephthalate-based and related carboxylate ligands	Porous Zr–carboxylate frameworks often regarded as relatively robust; biomedical systems are commonly engineered at the nanoscale, but colloidal behavior depends strongly on surface modification	Frequently used because of relatively high structural robustness, tunable porosity, and versatility in cargo loading and surface engineering; attractive for imaging and delivery applications	Although often regarded as stable, physiological phosphate sensitivity and formulation dependence remain important; strong framework stability may also complicate complete degradation or clearance	[25,27,32]
Fe-based MOFs	MIL-100(Fe), MIL-88, MIL-53, MIL-127 and related Fe-coordination systems	Fe	Carboxylate-based ligands, including trimesate, terephthalate, fumarate, and related derivatives	Meso- or microporous Fe-carboxylate frameworks with highly variable particle size depending on synthesis route, coating, and post-processing; accurate particle-size control is particularly important in biomedical settings	Broad biomedical interest due to iron bio-relevance, redox activity, drug-loading capacity, imaging potential, and reported biodegradability/biocompatibility	Redox activity and framework-dependent degradation pathways may complicate interpretation of efficacy, biosafety, and in vivo fate; particle-size control is particularly important for biomedical use	[27,33,34,35,36]
Porphyrinic MOFs	PCN-222, PCN-224 and related TCPP-based MOFs	Zr, Hf, Cu, Fe	TCPP and related porphyrinic ligands	Porous theranostic frameworks commonly engineered at the nanoscale; particle size, surface charge, and colloidal behavior may vary substantially after hybridization, surface decoration, or shell growth	Widely studied for photodynamic therapy, imaging, and theranostic applications because the ligand can contribute intrinsic photophysical functionality	Ligand-associated photodynamic, protein-binding, or other biological effects should be distinguished from metal-node or framework effects when possible	[27,32,37,38,39]
Cu-based MOFs (including HKUST-1-related systems)	HKUST-1, Cu-TCPP and related Cu-MOFs	Cu	BTC, TCPP, and related carboxylates	Porosity and dispersion behavior are framework-dependent; particle size and zeta potential are often highly medium- and coating-dependent in biomedical formulations	Explored in catalytic, antibacterial, sensing, wound-healing, and therapeutic contexts; HKUST-1 and Cu-TCPP are among the most discussed representatives	Copper release, redox activity, oxidative effects, and medium stability may narrow the practical safety window and complicate translational interpretation	[33,37]
CD-MOFs	γ-CD-MOF (CD-MOF-1) and related α/β/γ-CD-based frameworks	Mainly K^+^, Na^+^, Cs^+^; some other metal-ion variants reported	Cyclodextrin-based ligand frameworks	Host–guest inclusion behavior is more central than classical transition-metal pore interpretation; particle format and reported surface-charge data are comparatively formulation-specific	Particularly relevant to pharmaceutics and oral delivery because of edible/low-toxicity building blocks, host–guest inclusion capability, and potential to improve drug solubility, safety, and bioavailability	Structurally and translationally distinct from classical transition-metal nanoMOFs; should usually be interpreted separately from systemic metal-node nanomedicine platforms when discussing degradation and in vivo fate	[22,29]

The families summarized here are intended as representative framework classes relevant to biomedical discussion. Exact pore size, particle dimensions, and zeta potential vary substantially across specific frameworks, synthetic conditions, cargo loading, and surface-engineering strategies. Representative framework-level physicochemical parameters are therefore provided separately in Table S1. MOF platforms are now being explored in increasingly diverse in vivo settings. Yet development is still discussed mainly in terms of functional performance. Safety, in vivo fate, and degradation are less often considered as part of the same evaluation [3,24,40]. For formulations intended for in vivo delivery, outcomes such as tumor inhibition, imaging capability, or stimulus-responsive release are clearly relevant. On their own, however, these findings are rarely sufficient to support further development [3,24,41]. A fuller assessment also requires attention to where the material distributes after administration, how long it persists, how it is cleared, when the framework begins to disassemble, and whether released components may contribute to additional exposure or delayed risk [3,24,40].

**Table 2:** Four-Level MES Grading System for MOF Nanomedicines.

MES Level	Safety Evidence	In Vivo Fate Evidence	Degradation Evidence	Translational Meaning
1	Cytotoxicity, hemolysis, basic immune screening	—	Stability in buffered media; qualitative disassembly	Preliminary exclusion of clearly unsuitable materials
2	Single-dose tolerability; basic blood biochemistry; gross or histological observation of major organs	Qualitative organ distribution	Stimulus-responsive ion release under pH- or enzyme-related conditions	Helps judge whether the formulation merits further study but remains insufficient for translational interpretation of most systemic formulations
3	Short-term multi-organ toxicity, with dose context	Quantitative biodistribution and pharmacokinetic parameters	Mechanistic degradation in serum, PBS, or related media, together with drug release	Provides a basis for translational discussion, with interpretation dependent on the delivery context
4	Repeat-dose toxicity; long-term follow-up; immune-toxicity indicators	Quantitative excretion in urine, bile, or feces; mass-balance data	Metabolic fate of metal components and ligands; long-term retention	Supports development-oriented judgment closer to regulatory communication; long-term retention is generally a concern for systemic administration but may be functionally desirable for local platforms if local safety and controlled degradation are demonstrated

The translational meaning of the same MES criterion may differ depending on the delivery context. For systemically administered formulations, prolonged retention generally raises safety concerns because it may indicate incomplete clearance or long-term accumulation. For locally delivered systems, such as scaffolds, implants, or depots, prolonged residence may instead be desirable if local biocompatibility and controlled degradation are demonstrated.

**Table 3:** Key evidence limitations at lower MES levels and remaining gaps in representative Level 3 studies.

Category	Key Point	Main Implication
MES Level 1–2 studies	Fragmented evidence chain	Safety, in vivo fate, and degradation are commonly reported separately rather than as an integrated translational package.
	Safety limitations	Evidence often remains limited to cytotoxicity, hemolysis, single-dose tolerability, or short-window histology.
	In vivo fate limitations	Fate-related evidence is often qualitative, with limited pharmacokinetic, excretion, or mass-balance information.
	Degradation limitations	Degradation is frequently inferred from pH-responsive release or buffered-medium disassembly without mechanistic evaluation under physiologically relevant conditions [63].
MES Level 3 studies	Main remaining gaps	Repeat-dose toxicity, quantitative excretion, whole-body mass balance, and dual-component fate tracking are still commonly lacking.
MES Level 4	Current status	Studies approaching Level 4 remain rare or were not clearly identified in the current screened pool.

**Table 4:** Representative MOF ligand families and selected safety-relevant considerations.

Ligand Family	Representative Examples	Selected Evidence	Safety-Relevant Interpretation	Reference
Imidazole-based ligands	imidazole, 2-methylimidazole, ZIF linkers	ZIF degradation can release imidazolate species; in rats, 2-methylimidazole is rapidly absorbed and predominantly excreted in urine, with a large fraction recovered as parent compound. Evidence from methylimidazole literature should be interpreted cautiously across positional isomers.	Particularly relevant for degradable ZIF systems; released linker should be considered when interpreting safety findings.	[70,71,72]
Terephthalate-based ligands	BDC, NH2-BDC	Direct mammalian toxicokinetic data and benchmark toxicological evidence remain limited in the current MOF-relevant literature; available comparative evidence nevertheless suggests that ligand identity can influence biological responses.	Terephthalate-based ligands, especially substituted derivatives, should not be assumed biologically equivalent by default.	[73]
Porphyrinic ligands	TCPP and related porphyrinic linkers	Porphyrin-related species are biologically active and have been linked to protein interaction, oxidative injury, and transporter-regulated homeostasis.	Ligand-associated effects may contribute to both safety and fate readouts and should be distinguished from metal-node effects when possible.	[74,75,76]

**Table 5:** Representative original studies highlighting translationally relevant safety, in vivo fate, degradation/stability-related evidence, and exposure context in MOF platforms.

Study/Platform	Administration Context	Representative Dose/ Exposure Conditions	Key Safety Evidence	Key In Vivo Fate Evidence	Key Degradation/ Stability Evidence	Main Translational Limitation	References
MIL-127 oral detoxification platform	Oral/gastrointestinal-localized	Oral salicylate-overdose setting; simulated gastric (2 h, pH 1.2) and intestinal (24 h, pH 6.0) exposure; ex vivo testing at 1 mg mL^−1^	Oral safety and histological protection were reported	Poor intestinal permeation; GI confinement with fecal excretion	Strong GI stability with <9% degradation	Not a systemic nanomedicine fate study	[43]
Intranasal ZIF-8/Lip@Z slow-release platform	Intranasal/mucosal	Repeated intranasal dosing in mice (50–1000 μg); Lip@Z tested at 1000 μg; residence followed to 18 h	No significant serum, lung-function, or histology abnormalities versus saline	ZIF-8 coating prolonged nasal residence (half-life ~9 h vs. ~2.1 h for free liposomes)	Mucosal degradation was consistent with gradual local release/clearance	Local retention model, not a systemic disposition study	[42]
HSA-functionalized Hb@ZIF-8 oxygen carrier	Systemic oxygen-carrier concept	In vitro biocompatibility and physiological-media stability context; RAW 264.7 cells exposed to 0–5 mg mL^−1^ for 24 h	Improved macrophage biocompatibility and reduced opsonin-related adsorption	No robust whole-body in vivo fate package	HSA coating improved colloidal stability in physiological media	In vivo biodistribution and long-term safety remain unresolved	[46]
89Zr-UiO-66/Py-PGA-PEG-F3	Systemic tumor targeting/PET-tracked delivery	Intravenous PET imaging in orthotopic tumor-bearing mice; blocking dose ~10 mg kg^−1^; follow-up to 5 days	Histology and serum biochemistry supported absence of significant acute/chronic toxicity	PET organ distribution, ex vivo biodistribution, and tumor targeting were demonstrated	Excellent radiochemical and material stability in biological media	Excretion and full mass balance were not fully resolved	[45]
Renal-clearable Fe-CPNDs	Systemic tumor theranostic	Intravenous theranostic setting; hydrodynamic diameter ≈ 5.3 nm; clearance evaluated within 24 h	In vivo safety reported	Tumor accumulation and complete renal clearance within 24 h were reported	pH-activatable system with favorable colloidal stability	Long-term retention and organ-wide fate remain limited	[57]
In situ AFM degradation study of iron carboxylate MOFs	Mechanistic/non-administration-focused	Real-time degradation monitoring in PBS at neutral and acidic pH	Not the primary focus	No in vivo fate evidence	Surface erosion depended on crystal quality, defects, medium, and pH	In vivo linkage is absent	[63]
Quantitative guest-leakage study of ZIF-8 in physiological media	Mechanistic/physiological-media stability study	ZIF-8 tested in PBS, FBS, and albumin-containing media; guest leakage monitored by EPR	Not the primary focus	No in vivo fate evidence	Direct quantitative guest leakage was shown, with albumin-dependent instability highlighted	In vivo validation and carrier–cargo fate linkage are lacking	[94]
Surface-PEGylated UiO-66	Carrier/surface-engineering study	In vitro release and uptake setting; ~200 nm particles; pH 7.4 vs. 5.5 release comparison	In vitro biocompatibility context only	No in vivo fate evidence	PEGylation improved phosphate stability, reduced burst release, and enhanced pH-responsive release	No animal biodistribution or safety data	[67]
UiO-66@DOPA-LB long-circulating Zr-MOF nanoprobe	Systemic imaging/delivery	Intravenous administration in tumor-bearing mice; blood retention and tumor imaging followed to 24 h	Some in vivo safety support	Prolonged circulation and improved tumor accumulation were reported	DOPA coating improved stability under physiological phosphate challenge	Excretion and long-term organ burden remain insufficiently defined	[64]
Antibody-cloaked MOF-808 targeted delivery platform	Surface-engineered targeted delivery platform	In vivo imaging/tumor-inhibition setting in 4T1 xenografts with IR-780-loaded EGFR-M808	Safety was not the primary focus	Tumor accumulation and enhanced targeting were supported by imaging and tumor-inhibition experiments	Antibody precoating reduced protein adhesion and biomolecular corona formation	Comprehensive pharmacokinetic and long-term fate data are lacking	[69]
MIL-127/CS@MIL-127 intestinal-crossing study	Oral/intestinal-crossing study	Oral biorelevant exposure; *Caenorhabditis elegans* 24 h ingestion model; ex vivo rat intestinal crossing over 2 h	Biocompatibility was supported in vivo and ex vivo	Intact nanoMOF intestinal crossing was demonstrated, including rapid ex vivo crossing	Surface engineering affected aggregation, colloidal behavior, and oral-condition stability	Not a complete systemic disposition or excretion study	[44]

Dose or exposure descriptors are provided in a representative and context-dependent manner. Direct cross-study comparison remains limited because administration route, formulation design, payload, biological model, and reporting conventions vary substantially across studies. Mechanistic studies are included to support interpretation of degradation/stability-related evidence but do not constitute complete in vivo safety or disposition packages.

## Data Availability

No new data were created or analyzed in this study.

## Supplementary Materials

The following supporting information can be downloaded at: https://www.mdpi.com/article/10.3390/pharmaceutics18050548/s1. Table S1: Representative physicochemical parameters of selected MOF platforms commonly discussed in biomedical applications; Table S2: Detailed full-text MES coding of the curated MOF evidence subset. Additional study-level references supporting Table S2 have been incorporated into the main reference list [111,112,113,114,115,116,117,118,119].

## Abbreviations

The following abbreviations are used in this manuscript:

AFM	atomic force microscopy
BDC	benzene-1,4-dicarboxylate
BTC	benzene-1,3,5-tricarboxylate
CCDC	Cambridge Crystallographic Data Centre
CD-MOF	cyclodextrin-based metal–organic framework
CMC	chemistry, manufacturing, and controls
CSD	Cambridge Structural Database
CW EPR	continuous-wave electron paramagnetic resonance
DLS	dynamic light scattering
DOPA	dioleoylphosphatidic acid
EGFR	epidermal growth factor receptor
EPR	electron paramagnetic resonance
FBS	fetal bovine serum
GI	gastrointestinal
HA	hyaluronic acid
HSA	human serum albumin
ICP-MS	inductively coupled plasma mass spectrometry
IND	investigational new drug
LC-MS/MS	liquid chromatography–tandem mass spectrometry
MES	material evaluation and substantiation
MOF	metal–organic framework
NMOF	nanoscale metal–organic framework
PBS	phosphate-buffered saline
PDI	polydispersity index
PDT	photodynamic therapy
PEG	polyethylene glycol
PET	positron emission tomography
PK	pharmacokinetics
ROS	reactive oxygen species
SEM	scanning electron microscopy
TCPP	tetrakis(4-carboxyphenyl)porphyrin
TEM	transmission electron microscopy
TME	tumor microenvironment
UiO	Universitetet i Oslo
ZIF	zeolitic imidazolate framework
