# Supplementary material for: Safety, In Vivo Fate, and Degradation of MOF Nanomedicines: Toward Translational Evaluation
Source: Pharmaceutics. 2026 Apr 29;18(5):548. doi: 10.3390/pharmaceutics18050548 (PMC13211106; doi:10.3390/pharmaceutics18050548)
Supplement: Supplementary file 1 [file pharmaceutics-18-00548-s001.zip › pharmaceutics-4225457-supplementary.pdf]

Table S1. Representative physicochemical parameters of selected MOF platforms commonly discussed in biomedical applications.

MOF family	Representative framework	Representative metal/ligand	Typical pore size/aperture	Representative particle size reported in biomedical studies	Representative zeta potential	Notes on variability/interpretation	References
ZIF-type MOFs	ZIF-8	Zn/2-methylimidazole	Pore diameter ~11.6 Å; aperture ~3.4 Å	Frequently reported from tens to a few hundred nanometers, depending on synthesis and biomineralization conditions	NR for a stable benchmark value in the currently reviewed full texts	ZIF-8 is highly condition-sensitive in biological media; phosphate- and protein-containing environments can accelerate decomposition and alter apparent colloidal behavior	[28]
Zr-based MOFs	UiO-66	Zr/BDC (terephthalate)	Pore-size distribution ~8 and 11 Å	~100–200 nm for UiO-66-L1 by SEM; after PEGylation, average sizes of 160.2 ± 26.9 nm (PEG550) and 172.9 ± 36.8 nm (PEG2000) were reported	NR in the currently reviewed full texts	Dry-particle and hydrodynamic sizes may differ substantially; external coating strongly influences colloidal behavior	[25,27,32]
Zr-based MOFs	MOF-808	Zr/BTC	Large-pore Zr-carboxylate framework; a single benchmark pore-size value was not conservatively extractable from the currently reviewed full texts	In the antibody-cloaking study, pristine MOF-808 showed ~50 ± 10 nm, whereas IgG-coated particles showed ~150 ± 20 nm hydrodynamic size	−23.1 ± 3 mV for MOF-808 and −5.2 ± 1 mV after IgG coating	Surface defect chemistry and protein conjugation strongly influence biological-interface behavior; formulation-specific interpretation is required	[25,27,32]
Fe-based MOFs	MIL-100(Fe) nanoparticles	Fe/BTC	Mesoporous; a single benchmark pore diameter was not safely fixed from the currently reviewed texts	In the capsule study, preformed MIL-100(Fe) NPs showed 80 ± 30 nm by DLS and 66 ± 12 nm by TEM	−24 ± 0.6 mV in deionized water for preformed MIL-100(Fe) NPs	Particle size varies strongly with synthesis route, post-processing, and measurement mode; values from bare NPs should not be conflated	[27,33,35]

						with capsule or polymer-coated systems	
Fe-based MOFs	MIL-100(Fe) sub-micrometric capsules	Fe/BTC	Capsule porosity was preserved relative to the parent nanoparticles; micropore volume 0.64 $cm^3\ g^{-1}$ and mesopore volume 0.85 $cm^3\ g^{-1}$ were reported	Capsules showed an average diameter of ~800 nm; constitutive MIL-100(Fe) nanoparticles on the capsule surface were ~70 nm	NR for capsules	This is a hierarchical spray-dried capsule system rather than a simple nanoparticle benchmark and should be interpreted separately	[33,35]
Fe-based MOFs	HF-free MIL-100(Fe)	Fe/BTC	Surface area 1456.10 $m^2\ g^{-1}$ and pore volume 1.25 $cm^3\ g^{-1}$ were reported; a direct pore-diameter value was not extracted from the reviewed pages	Particles were described as octahedron-shaped	NR	Useful as a drug-delivery-oriented benchmark for surface area and pore volume, but not for a zeta-potential benchmark based on the currently reviewed pages	[33,36]
Fe-based MOFs	MIL-100(Fe)/CS	Fe/BTC with chitosan coating	NR for a stable pore-size benchmark in the currently reviewed pages	Chitosan coating was reported to increase particle size; exact final benchmark value should be locked only after full DLS/size figure confirmation	Chitosan coating was reported to alter surface charge; exact benchmark value should be locked only after full zeta figure confirmation	Best treated as a coated formulation rather than a bare-framework benchmark; polymer coating changes both colloidal behavior and release profile	[33,111]
Fe-based MOFs	MIL-127	Fe/azobenzenetetracarboxylate derivative	One-dimensional channels ~6 Å and cages ~10 Å, accessible through narrow apertures of ~3 Å	Donor-chamber hydrodynamic size was ~214 nm for MIL-127 and ~392 nm for CS@MIL-127; crossed nanoMOFs were ~150 nm	About −23 mV (MIL-127) and −24 mV (CS@MIL-127) in GI-relevant media	Physicochemical behavior is strongly medium-dependent; chitosan coating changes aggregation and chemical stability	[27,33]
Porphyrinic MOFs	PCN-224	Zr/TCPP	A single benchmark pore-size value was not conservatively fixed from the	In the PCN-224@Co3O4-HA study, pristine PCN-224 was ~180 nm; in the PCN-224@ZIF-8 study,	In the PCN-224@Co3O4-HA study, pristine PCN-224 was +12 mV	Reported particle size is strongly preparation-specific; PCN-224 values should be interpreted in formulation context rather	[27,32,37,38,39]

			currently reviewed full texts	pristine PCN-224 was ~110 nm and ~107.5 nm in PBS		than as a universal intrinsic size	
Porphyrinic MOFs	PCN-224@Co3O4-HA	Zr/TCPP with HA-modified Co3O4 decoration	NR for a stable pore-size number in the reviewed pages	Composite particle size was ~200 nm	Zeta potential changed from +12 mV to −27 mV after HA-Co3O4 integration	This is a targeted composite nanoplatform rather than a bare framework benchmark; the negative surface charge reflects HA-modified Co3O4 integration	[37,38]
Porphyrinic/ZIF hybrid MOFs	PCN-224@ZIF-8	Zr/TCPP core with Zn/2-methylimidazole shell	Pore size remained nearly unchanged relative to PCN-224 according to the paper; an exact numeric pore-size value was not given in the reviewed pages	Average diameters were ~110 nm for PCN-224 and ~150 nm for PCN-224@ZIF-8; in PBS they were ~107.5 nm and ~155.5 nm, respectively	Zeta potential was reported to be significantly lower than that of PCN-224, but the exact value was not given in the reviewed text	BET surface area increased from 353.03 $m^2 g^{-1}$ (PCN-224) to 361.34 $m^2 g^{-1}$ (PCN-224@ZIF-8); this hybrid should be treated as a composite benchmark rather than a pure PCN-224 benchmark	[37,39]
Cu-based MOFs	HKUST-1	Cu/BTC	Pore size ~15.9 Å in the Cu-MOF review	Nanoscale HKUST-1 examples in the currently reviewed texts included ~70 nm and a tunable range of 24–567 nm	NR in the currently reviewed full texts	Particle size is highly synthesis-dependent; copper release and redox reactivity may be more important than nominal framework parameters in biomedical interpretation	[34,37]
CD-MOFs	γ-CD-MOF (CD-MOF-1)	K^+/γ-cyclodextrin	γ-CD cavity/aperture up to ~1.69 nm	NR for a stable benchmark particle size in the currently reviewed texts	NR	Best interpreted separately from classical transition-metal nanoMOFs; host–guest inclusion behavior and oral/pharmaceutics relevance are more central	[22,29]

Physicochemical parameters are provided in a representative manner for selected benchmark frameworks frequently discussed in biomedical contexts. Reported pore size, particle dimensions, and zeta potential vary substantially depending on synthetic route, particle-size metric (e.g., dry-particle vs hydrodynamic size), cargo loading, surface engineering, and dispersion medium.

Table S2. Detailed full-text MES coding of the curated MOF evidence subset.

MES level	Pool	Delivery context	Safety evidence	In vivo fate evidence	Degradation/stability evidence	Main missing evidence for upgrade	Notes	Reference
L3	Primary	Systemic tumor theranostic	In vivo safety reported	Tumor accumulation and renal clearance within 24 h	pH-related disassembly evidence	Organ-wide time course, mass balance, long-term safety	Strong fate-oriented study	[57]
L3	Primary	Systemic TNBC therapy	30-day toxicity, hematology/biochemistry, H&E	Organ Zn burden assessed	ATP/pH-responsive degradation and release	Early biodistribution, excretion route, ligand-resolved fate	Strong systemic evidence	[60]
L3	Primary	Systemic multimodal tumor therapy	In vivo safety/body weight/H&E	Main-organ Fe/Cu distribution reported	Biodegradable framework with imaging-supported degradation	Excretion quantitation, component-resolved fate	Primary example	[91]
L3	Primary	Systemic stabilized MOF delivery	Acceptable in vitro/in vivo biocompatibility package	Limited hard whole-body fate package, but improved physiological stability supports stronger translational interpretation	In situ polymerization enhanced physiological stability while preserving stimulus-responsive intracellular drug delivery	Quantitative biodistribution, excretion, mass balance, and long-term retention	Strong stability-oriented primary study	[61]
L3	Primary	Systemic theranostic	Comparative in vivo evaluation	Quantitative 64Cu PET; size-dependent circulation/tumor uptake	pH-responsive dissolution/release	Excretion, long-term organ burden	Strong primary paper	[58]
L3	Primary	Systemic radiolabelled/biodistribution-oriented MOF study	Toxicity, stability, radiolabelling and biodistribution addressed	Radiolabelled biodistribution reported	Framework stability discussed	Long-term fate, excretion, component-resolved fate	primary paper	[59]
L3	Primary	Systemic PET-tracked UiO-66	Acute/chronic toxicity, histology, biochemistry	PET organ distribution and ex vivo biodistribution	Serum/material stability improved by PEGylation	Excretion, mass balance	Strong primary paper	[45]
L3	Primary	Systemic polymer-shielded ZrMOF	Some safety support	64Cu PET, prolonged circulation, improved tumor accumulation	Strong stabilization/degradation engineering	Excretion, mass balance	Primary paper	[64]
L3	Primary	Systemic lanthanide theranostic/real-time imaging	Systemic theranostic package	Tumor-responsive imaging/in vivo tracking evidence	Bidirectional near-infrared photon-conversion-associated responsive behavior	Quantitative excretion, long-term retention, component-resolved fate	Strong primary paper	[62]

L3	Primary	Systemic AAA nanomedicine	Favorable biosafety	Effective accumulation at AAA site with prolonged retention	pH/ROS-responsive release; improved colloidal stability	Organ-wide quantitation, excretion, component fate	Strong primary paper	[54]
L3	Primary	BBB-penetrating targeted stroke nanozyme	Strong neuroprotective/safety package	BBB transport and ischemic lesion accumulation	H_2O_2-responsive release plus ROS scavenging	Excretion, component-resolved fate	Strong primary paper	[112]
L2–3	Primary	Systemic MDR tumor therapy	Body weight/H&E/biochemistry	No robust whole-body fate dataset	Acid-sensitive ZIF-8 shell degradation	Biodistribution, clearance, component fate	Transitional primary study	[87]
L2–3	Primary	Systemic tumor therapy/imaging	Good biocompatibility claims	Limited qualitative metabolism/fate	In vitro degradation shown	Quantitative biodistribution/excretion	Transitional primary study	[90]
L2–3	Primary	Systemic chemo/PTT theranostic	Histology/major-organ safety	Limited imaging/accumulation evidence	Acid-triggered outer-shell degradation	Quantitative fate and clearance	Transitional primary study	[85]
L2–3	Primary	Systemic Bio-MOF/PDA targeted theranostic	Moderate/acceptable biocompatibility	Targeting logic present, but limited hard fate data	Improved physiological stability; multi-drug chemo-photothermal response	Quantitative fate and long-term safety	Transitional primary study	[113]
L2–3	Primary	Systemic redox-dyshomeostasis tumor therapy	Good in vivo tolerability	In vivo tumor study, but incomplete fate package	Stimulus-responsive redox-related disassembly/release logic	Biodistribution, excretion	Transitional primary study	[114]
L2–3	Primary	Systemic multifunctional MOF nanosystem for synergistic therapy	Good in vitro/in vivo biocompatibility	Limited hard fate dataset	Redox-homeostasis disruption with stimulus-responsive disassembly/release	Biodistribution/excretion, long-term fate	Transitional primary study	[115]
L2	Contextual	Local cardiovascular stent coating	Local hemocompatibility and implant biocompatibility	No systemic fate	Cu release/local stability only	Systemic biodistribution, clearance, long-term retention	Contextual device/local study	[66]
L2	Contextual	Targeted systemic delivery	Some in vivo application evidence	Limited	Not degradation-focused	Quantitative fate, degradation chain	Contextual targeting paper	[69]
L2	Contextual	Therapy platform; ultimately intratumoral use	Some hemolysis/in vivo data	Liver/spleen accumulation only; no full fate	pH-responsive release	Systemic fate and clearance	Contextual application study	[77]
L2	Contextual	Bone-targeted immunotherapy	In vivo therapeutic evidence	Bone targeting only; no full fate	Acid-triggered release logic	Organ distribution/excretion	Contextual targeting paper	[106]

L2	Contextual	Oral detoxification	In vivo oral safety	Poor intestinal permeation	Strong GI stability	Not a systemic nanomedicine fate study	Contextual oral-retention case	[43]
L2	Contextual	Intra-articular OA nanogel	Biosafety plus local repair	Local retention rather than systemic fate	Sustained release over one month	Systemic biodistribution/excretion	Contextual local-delivery study	[51]
L1–2	Contextual	Oral/physiological release concept	Limited	No	Framework dissolution/release in media	Any in vivo safety/fate	Contextual release study	[106]
L1–2	Contextual	Mechanistic degradation study	Not primary focus	No	Strong mechanistic degradation evidence	In vivo linkage	Mechanistic support	[63]
L1–2	Contextual	Ligand-mediated protein corona/structure-protein-cell interaction	In vitro cytotoxicity-associated evidence	No	Not degradation-focused	Animal safety/fate	Contextual stability paper	[98]
L1–2	Contextual	Hb@ZIF-8 oxygen carrier	Some in vitro biocompatibility	No robust in vivo fate	Stability/corona stealth effects	In vivo distribution and safety	Contextual carrier study	[46]
L1–2	Contextual	Local periodontitis hydrogel-MOF	In vitro/in vivo local repair	No systemic fate	Controlled local release/hydrogel-supported stability	Systemic safety/fate	Contextual local-delivery study	[52]
L1–2	Contextual	Local oral-ulcer microneedles	Good local therapeutic effect	No systemic fate	Slow biodegradable local release	Systemic fate	Contextual local-delivery study	[53]
L1–2	Contextual	MOF-derived nanozyme for myocardial injury	Therapeutic benefit shown	No robust whole-body fate	Stability only partially addressed	PK/distribution/excretion	Contextual nanozyme paper	[116]
L1–2	Contextual	Ligand-screened Ce-based MOF microcapsules for nerve regeneration	Local regenerative evidence	No systemic whole-body fate	Local release/microcapsule-supported stability	Systemic biodistribution, clearance	Contextual regenerative study	[117]
L1–2	Contextual	Local SCI hydrogel/H2S-Zn MOF	Good local repair package	No systemic fate	ROS-triggered H2S/Zn^{2+} release	Systemic fate chain	Contextual local-delivery study	[55]

L1–2	Contextual	Exosome-functionalized Mg-GA scaffold	Good local bone-regeneration evidence	No systemic fate	Slow exosome/Mg^{2+}/GA release	Systemic fate	Contextual local-delivery study	[56]
L1–2	Contextual	Liposome/Fe-BTC dual-responsive carrier	Low cytotoxicity below tested range	No in vivo fate	pH/ultrasound dual-responsive release	Animal safety/fate	Contextual carrier study	[118]
L1	Contextual	Biocompatible/biodegradable rifampicin carrier	In vitro biocompatibility	No in vivo fate	Carrier/release study; complete release in PBS within hours	Any in vivo evidence	Contextual carrier study	[119]
L1	Contextual	Oral/local pH-responsive release concept	Very limited	No	Structure/release stability only	Any in vivo evidence	Contextual release study	[92]
L1	Contextual	Protein corona/uptake	In vitro only	No	Not degradation-focused	Any in vivo evidence	Biological-identity support	[96]
L1	Contextual	Ligand/corona/cell interaction	In vitro only	No	Not degradation-focused	Any in vivo evidence	Contextual interaction study	[98]

Table S2 provides detailed full-text, study-by-study MES coding for a curated subset of the literature. This subset was used for deeper evidence-chain interpretation and therefore differs from the broader screened original-study pool used for the overview-level MES distribution shown in Figure 1. Primary MES-mapping pool was restricted to studies most directly relevant to translational interpretation of nanomedicine-oriented evidence, whereas local scaffold-, hydrogel-, implant-, depot-, or microneedle-focused studies were retained as contextual evidence and not merged into the same interpretation framework.
